# The Robotic-Assisted Laparoscopy, Isthmusectomy, and Pyeloplasty in a Patient With Horseshoe Kidney: A Case Report

**DOI:** 10.1097/MD.0000000000002516

**Published:** 2016-01-15

**Authors:** Sheng Tai, Jianzhong Wang, Jun Zhou, Zongyao Hao, Haoqiang Shi, Yifei Zhang, Chaozhao Liang

**Affiliations:** From the Department of Urology, the First Affiliated Hospital of Anhui Medical University (ST, JW, JZ, ZH, HS, YZ, CL); and the Urological Institute of Anhui Medical University, Hefei, Anhui, PR China (ST, JW, JZ, ZH, HS, YZ, CL).

## Abstract

The aim of this case report was to evaluate the results of isthmusectomy and pyeloplasty of horseshoe kidney with the da Vinci robotic-assisted laparoscopy system.

This case presented 1 patient with left back pain, associated with lower abdominal pain, and then she underwent the isthmusectomy and dismembered pyeloplasty using robotic-assisted laparoscopy simultaneously. The operation was performed by a transperitoneal approach using 5 ports.

We cut the renal isthmus by means of bipolar scissors and then closed the renal parenchyma with 3–0 absorbed stitches. The total operation time was 123 min including simultaneous dismembered pyeloplasty. Blood loss was <50 mL. There were no complications either during or after the procedure. The oral nutrition and mobilization were included on the second day after surgery. The peritoneal drainage was removed on the eighth day. Long-term follow-up after treatment showed good results.

The da Vinci robotic-assisted laparoscopy is an alternative to open surgery and laparoscopy, particularly in the correction of congenital defects of the urinary tract. Furthermore, the da Vinci robotic-assisted laparoscopy technique in isthmusectomy and pyeloplasty is safe for patient as shown by our results.

## Abstract 2

Supplemental Digital Content is available in the text

## INTRODUCTION

Horseshoe kidney is one of the congenital urinary tract anomalies occurring 1 in 400 of the general population.^1^ Horseshoe kidney is the connection of lower, rarely, the upper renal pole. The renal pelvis faces the front, whereas the calyxes face the spine. The ureter extending from the front are entangled on the poles connecting the isthmus cause urine flow hindrance, which results in some causes to an expanded pyelocalyceal system and hydronephrosis.^1,2^ It has been reported that numerous additional vessels extending from and to the renal isthmus cross with the ureters, causing ureter obstruction.^3,4^ Urinary outflow obstruction causes urinary retention and formation of calculi in the horseshoe kidney. The ureteropelvic junction obstruction (UPJO) occurs in the 15% to 33% of patients with horseshoe kidney.^2,3,5^

The defect does not cause any symptoms and is often diagnosed incidentally during routine imaging exam, and some patients complain of abdominal pain but with an indefinite placement.^3,4^ Physicians performed the ultrasound imaging, intravenous pyelography (IVP), or computed tomography (CT) to identify the horseshoe. The indications for surgery are associated with horseshoe kidney abnormalities such as UPJO or nephrolithiasis.^1,2^ Currently, division of the horseshoe kidney isthmus is rarely done, and if it is done, it is during the simultaneous removal of additional defects accompanying horseshoe kidney.^1^ The operation of cutting vessels crossing the ureter in a patient with horseshoe kidney was described.^6^

It has been reported that the laparoscopic heminephrectomy has been done among patients with nonfunctional horseshoe kidney, or horseshoe kidney cancer.^3,7,8^ Certain studies showed the patients with horseshoe kidney and UPJO could undergo the laparoscopic isthmusectomy and dismembered pyeloplasty at the same time.^1,9^ The robotic laparoscopy, characteristic of short-term experience, short learning curve, and so on, makes these cases an increasingly feasible alternative to the laparoscopy surgery. In this case, we first present the results of isthmusectomy and dismembered pyeloplasty of horseshoe kidney with the da Vinic Surgical System all over the world.

## CASE PRESENTATION

**FIGURE 1:**
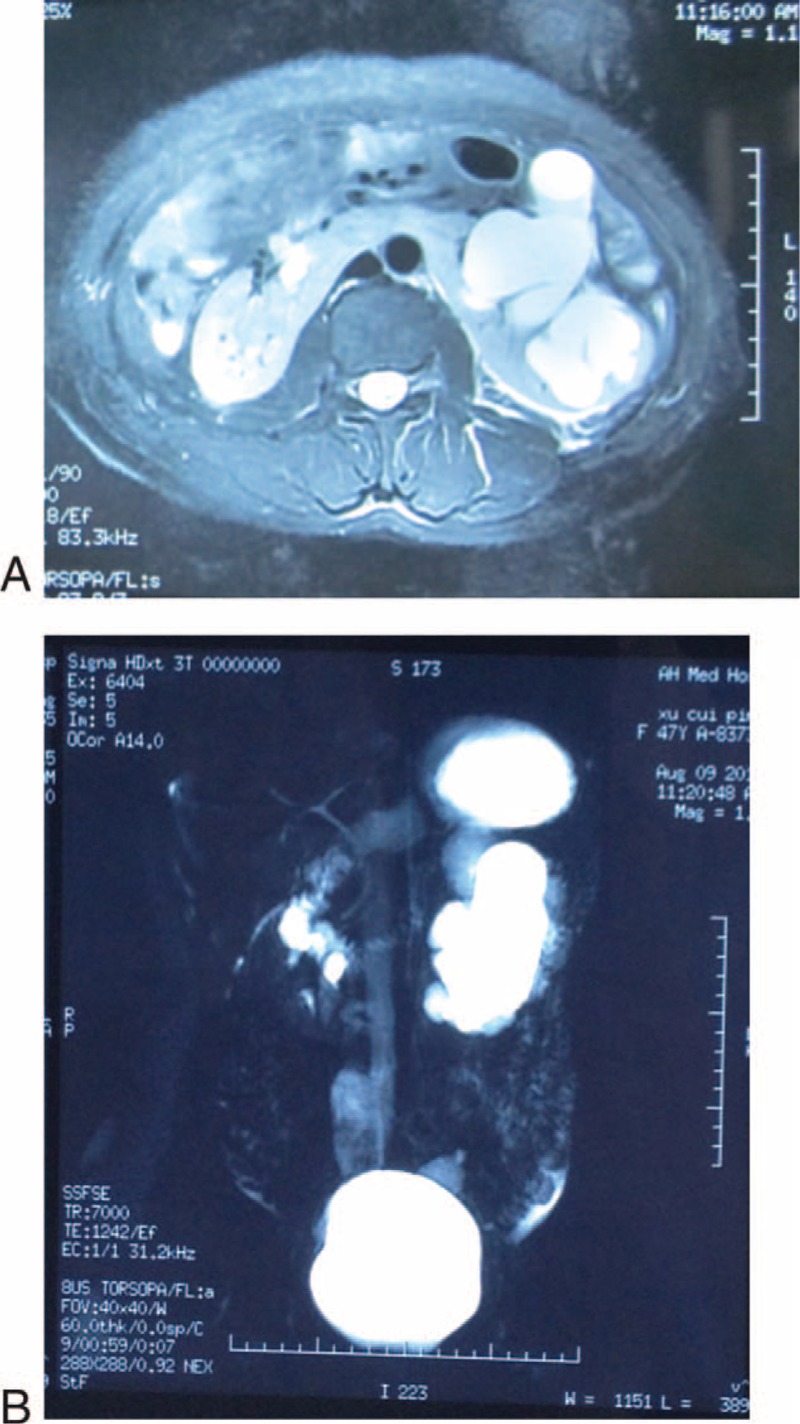
The MRI (T2) scan (A) showed the horseshoe kidney in this patient, and MRU (B) demonstrated the left kidney is hydronephrosis and UPJO. MRI = magnetic resonance imaging, MRU = magnetic resonance urography, UPJO = ureteropelvic junction obstruction.

One 47-year-old woman complained of left back and lower abdominal pain. This patient had not any renal stones. The magnetic resonance imaging and magnetic resonance urography scans demonstrated a horseshoe kidney with concomitant left UPJO and hydronephrosis (Figure 1A and B). This patient was no history of hematuria, urinary tract infection, and renal stones.

**FIGURE 2:**
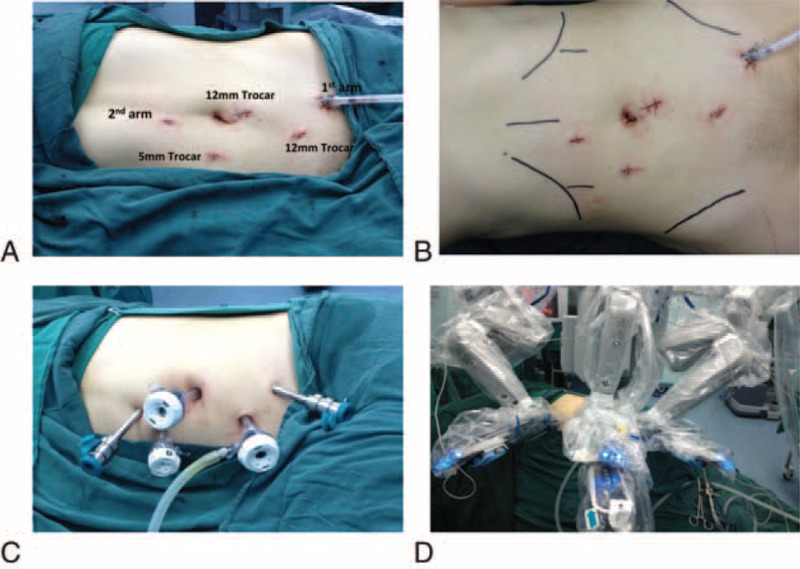
The patient was in a semilateral decubitus position and 5 ports position were illustrated in A, B, C. The da Vinci robotic-assisted laparoscopy system was assembled (D).

**FIGURE 3:**
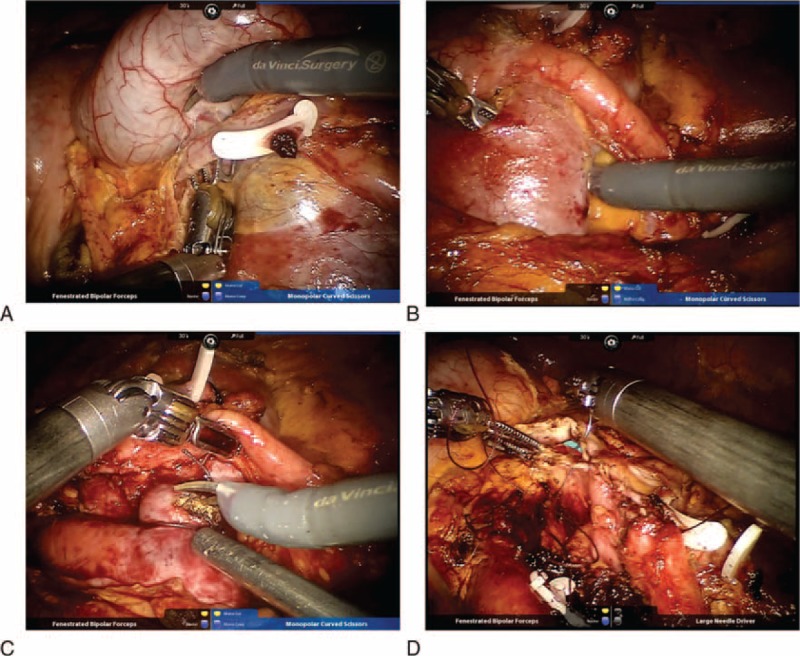
The ectopic and ovary veins were divided (A). The isthmus of horseshoe kidney was separated, stitched, and divided (B, C). The pyeloplasty was performed (D).

This patient was performed the trans-abdominal robotic laparoscopy. The patient was rolled into a semilateral decubitus position rotating the operative side up by 45°axially (Figure 2A). The 5 trocars placement was performed (Figure 2A and B). After the pneumoperitoneum was created, a 12-mm port was placed on the para-umbilical line for the robotic camera. For the robotic arms, 2 8-mm ports were placed subcostally in the left iliac fossa and on the right midclavicular line, respectively. One accessory 12-mm port and the other accessory 5-mm port were placed adjacent to the umbilicus. The lateral peritoneal reflection was incised, and then the decent and partial transverse colons were reflected medially. In order to expose the UPJ and isthmus, the Gerota's fascia was dissected. One ectopic renal vein crossed the UPJ, resulting in the UPJO. The ectopic renal and left ovary veins were divided and cut, respectively (Figure 3A [see Video, Supplemental Video, which demonstrates the details of procedure]). The isthmus connecting the lower pole of either kidney was isolated, and then certain vessels reaching the isthmus were divided. It is more feasibly and easily to divide, coagulate, and cut these tiny blood vessels with the robotic-assisted laparoscopy. None of them could cause ischemia of the parenchyma. The robotic dissector was placed under the isthmus when it was raised upward (Figure 3B and C [see Video, Supplemental Video, which demonstrates the details of procedure]). We sutured the isthmus from up to down by 2 separate 1–0 absorbed stitches, and then we cut the renal parenchyma between stitches using bipolar coagulation scissors. The renal parenchyma was stitched with 2 3–0 absorbed stitches on the edges of isthmus again. (See Video, Supplemental Video, which demonstrates the details of procedure.)

The redundant plevis and stenosis ureteric segment were both excised. The renal pelvis was reconstructed with a 4–0 absorbable running suture. An F6 double J stent was placed in an ante-grade manner, and then the posterior and anterior aspects of the pelvic-ureteric anastomosis were both done by the 4–0 absorbable running suture (Figure 3D). Finally, 1 drainage tube was placed (see Video, Supplemental Video, which demonstrates the details of procedure).

**FIGURE 4:**
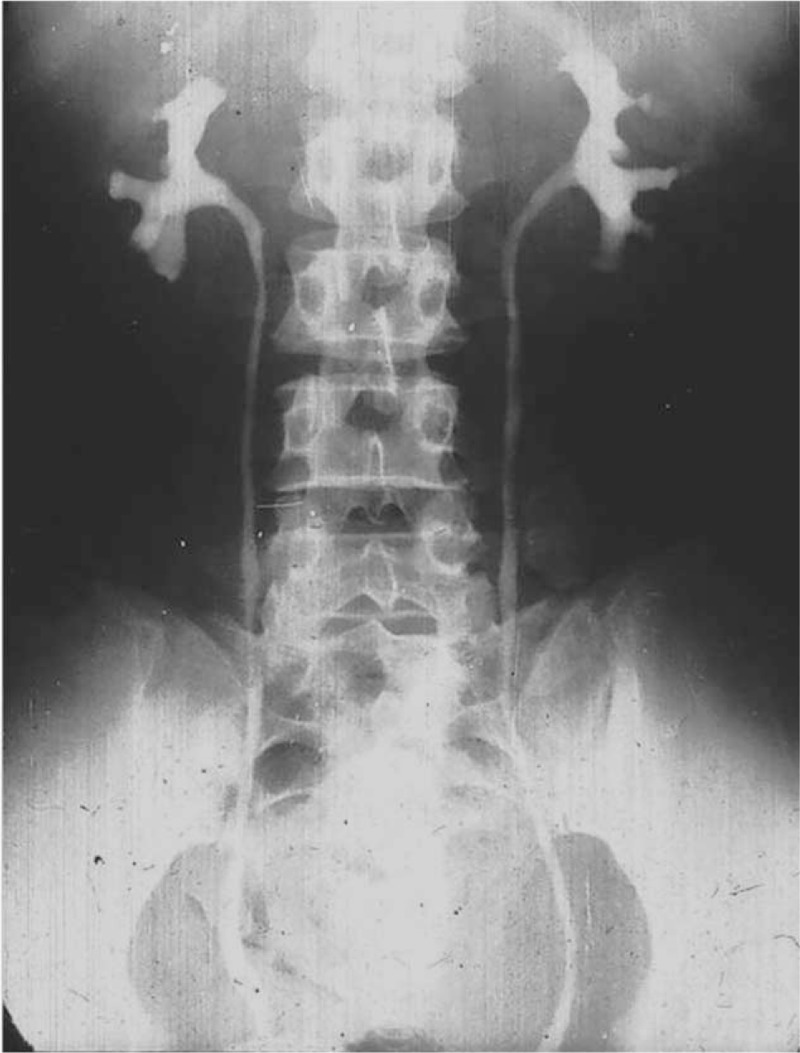
The IVP showed the good renal function and improved drainage at 11.5 months after surgery. IVP = intravenous pyelography.

All procedures were done successfully. The operative duration was 123 min and estimated blood loss was <50 mL. The patient took some food and activity on the second after surgery. The drainage tube was removed at 8 day, and hospital duration was 9 days. The double J stent was removed after 2 months. Following up, the IVP exam showed this female patient had good renal function and improved drainage at 11.5 months after surgery (Figure 4). The case report was approved of the Ethical Committee of the First Affiliated Hospital of Anhui Medical University (Hefei, China, EC20150612) and approved of the patient consent.

## DISCUSSION

Horseshoe kidney is a congenital defect, which occurs in 1 in 500 individuals. It is twice as often identified in men as in women. It is frequently accompanied by other anomalies of the pyelocalyceal system.^5,10,11^ It has been reported that the defects are associated with horseshoe kidney in 52% of the children with horseshoe kidney.^12^ Difficult urine outflow is due to the course of the ureter, which comes out anterior of the pelvis and crosses the isthmus of the kidney. In additional, the numerous vessels extending into the isthmus and lower pole may cross the ureter.^13^ In this patient, 1 ectopic renal vein crossed the left ureter. The consequence due to obstruction of the urine outflow may be destruction of the renal parenchyma. Meanwhile, it may also result in the formation of stones.^12^ Patients with horseshoe kidney may not display any symptoms. If there are some symptoms, the most frequently reported ones include abdominal pain, vomiting, and nausea.^2,14^ This female patient is diagnosed with horseshoe and UPJO by physical examination.

Horseshoe kidney with UPJO or stones was direct indications for surgery. For UPJO, the da Vinci robotic-assisted laparoscopic dismembered pyeloplasty has more superiority than laparoscopic surgery.^15,16^ The aim of the surgery was dismembered pyeloplasty and isthmusectomy. The da Vinci system has been characteristic of tremor filtering, movement scaling, enhanced dexterity, increasing range of motion, 3-dimensional visualization, and so on.^15^ Some studies showed that the da Vinci robotic-assisted laparoscopy may perform dismember pyeloplasty in patients with horseshoe kidney.^5^ Some other studies also demonstrated the laparoscopic heminephrectomy, nephrectomy, or isthmusectomy may be done in the horseshoe kidney.^3,4,10,11^ In this case, with da Vinci robotic-assisted laparoscopy system, we performed not only the dismembered pyeloplasty, but the isthmusectomy. To our knowledge, such a surgery technique has not been reported previously among the horseshoe kidney patient.

Certain urologists proposed to divide the isthmus, nephropexy, and then dismember pyeloplasty during the open surgery. The division may be done, with electrocautery, microwave coagulation, ultrasonic shears, and so on, under direct vision.^17^ Other studies demonstrated the Anderson-Hynes pyeloplasty could be enough, and it is unnecessary to divide the isthmus.^5^ Consistent with the open surgical strategy, the dismembered pyeloplasty and divided isthmus has been both done simultaneously in this case (see Video, Supplemental Video, which demonstrates the details of procedure), and 1-year follow-up witnessed the effective treatment results. Robotic pyeloplasty may cost more than standard laparoscopy, but its advantage may outweigh this. The isthmus is often in the midline, so it is difficult to divide it with traditional laparoscopy.^15^ The da Vinci robotic-assisted laparoscopy system provides the possibility that is more feasibly and easily to carry out the isthmusectomy and pyeloplasty among patient with horseshoe.

## CONCLUSION

Our initial experience suggests that the indications for robotic-assisted isthmusectomy and pyeloplasty can be extend to anatomically challenging case such as this patient with horseshoe kidney. The robotic laparoscopy system allows surgeons to perform these complex procedures easily, safely, and effectively.

## Supplementary Material

Supplemental Digital Content
